# Wear Characteristic of Stellite 6 Alloy Hardfacing Layer by Plasma Arc Surfacing Processes

**DOI:** 10.1155/2017/6097486

**Published:** 2017-11-20

**Authors:** Zhiyuan Zhu, Chun Ouyang, Yanxin Qiao, Xiaowei Zhou

**Affiliations:** School of Material Science and Engineering, Jiangsu University of Science and Technology, Jiangsu 212003, China

## Abstract

The microstructure and wear resistance of Stellite 6 alloy hardfacing layer at two different temperatures (room temperature and 300°C) were investigated by plasma arc surfacing processes on Q235 Steel. Tribological test was conducted to characterize the wear property. The microstructure of Stellite 6 alloy coating mainly consists of *α*-Co and (Cr, Fe)_7_C_3_ phases. The friction coefficient of Stellite 6 alloys fluctuates slightly under different loads at 300°C. The oxide layer is formed on the coating surface and serves as a special lubricant during the wear test. Abrasive wear is the dominant mechanism at room temperature, and microploughing and plasticity are the key wear mechanisms at 300°C.

## 1. Introduction

Cobalt- (Co-) based alloys (e.g., Stellite alloy) are widely used in the wear environment because of their good resistance to corrosion, wear, and abrasion [1]. Stellite systems are Co alloys that mainly contain alloying elements, such as tungsten (W), chromium (Cr), molybdenum (Mo), and a certain amount of carbon (C). Cr is the main alloying element that reacts with C to form interdendritic carbide. The alloying elements W and Mo also react with C to form carbide as secondary particles. Solid solution strengthening of Co-based alloys with carbides can be easily achieved. The distribution, size, and shape of carbides are determined by the processing condition and affect the mechanical properties and hardness [2–5].

Stellite 6 alloys coated on stainless steel are used for valve application in a high-temperature and high-pressure environment, where a material may melt, creep, or degrade. In our application, the working temperature is between 250°C and 300°C [6]. In such application, alloys play an important role in preventing various wear factors that affect the sliding surface. Oxygen in air is a normal element that reacts with alloying elements, particularly at high temperatures [7–10]. The formation of oxides plays an important role in the wear process [11]. The alloying element Cr shows good wear and corrosion resistance and high-temperature strength. Fontalvo and coworkers demonstrated that oxides formed at high temperatures reduced wear and served as protective film between the contacting areas [12, 13]. The wear performance of Stellite 6 alloys was relatively superior at 750°C. Oxides growing slowly on Stellite 6 alloys lead to the formation of a protective film with good wear resistance [14]. Wang and coworkers demonstrated that the oxide scale increased with the addition of yttrium to Stellite alloy to improve the wear performance at 650°C. When the surface of Stellite 6 alloy was subjected to wear, heat expansion, and oxidation, the oxide film was destroyed. The fresh surface was exposed, and oxidation was accelerated. Ultimately, the film was completely broken. This process was repeated, and the wear resistance decreased. Therefore, the bonding strength of the oxide film is a key factor affecting the total resistance at elevated temperature [15–22].

This study aims to evaluate the effect of temperature on wear resistance, which is the working temperature of the valve in real application. The process and mechanism of wear are discussed at room temperature and 300°C.

## 2. Experimental Procedure

### 2.1. Preparation of Stellite 6 Hardfacing

Q235 mild steel was used as substrate, and Stellite 6 alloys were welded by plasma transferred arc welding (PTAW). The composition (wt%) of Q235 mild steel was 0.16 C, 0.53 Mn, 0.30 Si, <0.045 P, <0.055 S, and balanced Fe. The composition of Stellite 6 powder is listed in Table 1. The surface of the matrix was cleaned with acetone and washed with distilled water to remove residue and grease. Afterward, the matrix was air-dried. The Stellite 6 powder was prepainted on the surface of the substrate. The thickness of powder was approximately 2 mm. The coating was prepared by the PTAW method with a current of 150 A.

### 2.2. Microstructural Characterization

Q235 mild steel with coating was cut along the perpendicular direction of the contacting line of the substrate and coating. Then, the cutting surface of the coating was enclosed with Bakelite. The surface of the specimens was polished by SiC sandpaper down to 1,500#, followed by polishing pad with alumina powder. Then, the surface was washed with distilled water and ultrasonicated in a water bath for 5 min. Afterward, the microstructure of the coating was etched by nitrohydrochloric acid. The images of the coating before and after wear were taken by an optical microscope and a scanning electron microscope (SEM). The morphology of coating taken from different spots was analyzed. X-ray diffraction (XRD) was used to characterize the phase analysis conducted using the XRD equipment with Cu K*α* radiation. A step of 0.02° was used to scan the 2*θ* degree from 30° to 100°. The specimen for XRD test was preetched in 10 wt% oxalic acid for 90 s at an anode polarization potential of 6 V at room temperature.

### 2.3. Test of Wear Resistance

The wear test was conducted using the UMT-2 Friction–Wear Tester (USA). Specimens with the dimensions of 15 mm × 15 mm × 4 mm were taken from the coating. The surface of the specimens was polished and cleaned with acetone and distilled water. The tribological test was conducted using the ball-on-disk tribometer with 10 N loading at different temperatures. The tests were conducted using C45 spherical steel (ASTM 1045) with a diameter of 9.38 mm. The speed of the specimen against the ball was 2.5 cm s^−1^ in 30 min. After the test, the morphology of the wear track was checked by SEM and confocal laser scanner (LEXTOLS400). Then, the wear rate was calculated using *Ws* = *CA*/*FL*, where *C* is the length of the wear track, *A* is the average area of wear loss, *F* is the loading, and *L* is the distance of the wear. After the experiments, the surface of the specimens was characterized by SEM.

## 3. Results and Discussion

### 3.1. XRD Analysis of Stellite 6 Alloys

**Figure 1:**
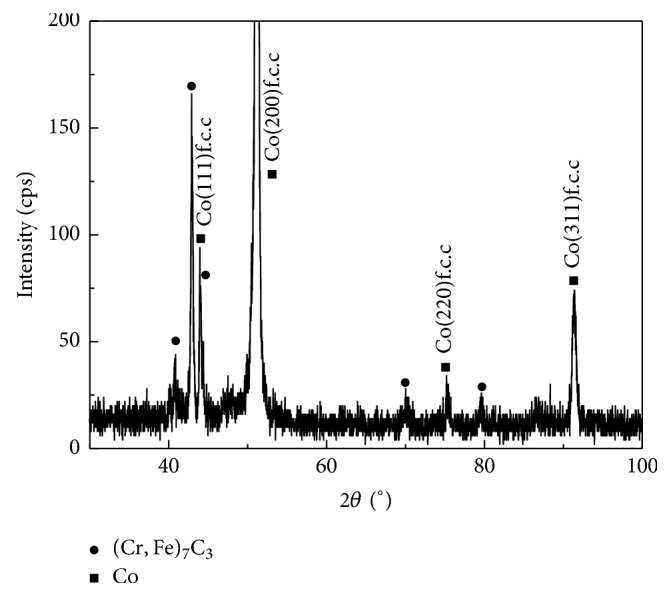
XRD pattern of the welding coating.

The results of XRD analysis of Stellite 6 alloy coating are shown in Figure 1. The phases of deposited coating are *α*-Co and M_7_C_6_ (M = Fe, Cr), which are determined by comparison with the lattice parameter of standard JCPDS cards. The highest peak intensity is recorded for the *α*-Co and (Cr, Fe)_7_C_3_ phases, and the peaks of these two phases overlap. The presence of (Cr, Fe)_7_C_3_ [23] plays a key role in increasing the hardness of the coating [24].

### 3.2. Microstructure of Stellite 6 Alloy Hardfacing

**Figure 2:**
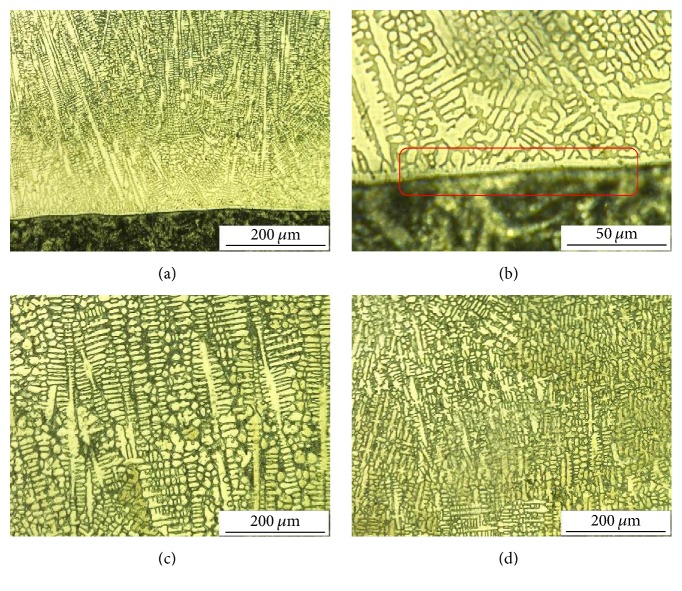
Microstructure of hardfacing layer: (a) fusion line, (b) dilution zone, (c) transition zone, and (d) fine crystalline zone.

Figure 2 shows the microstructure of the coating from substrate to surface. Generally, the microstructure of welding coating is divided into three parts, namely, dilution, transition, and fine grain zones [25].  Figure 2(a) shows the fusion line between substrate and coating. The dilution zone of the coating is shown as red rectangles in Figure 2(b). Planar crystalline structures were observed over the substrate. These planar crystalline structures come in contact with the grains of the substrate. New grains usually form from unmelted grain. The size of the new grains increases along the original direction of the crystal. Then, columnar crystal is formed along the perpendicular direction of the fusion line, as shown in Figure 2(c). As solidification proceeds, the upper part of the coating around the surface becomes the fine grain zone, as shown in Figure 2(d). In the fine grain regions, the crystal exhibits multidirectional growth.

**Figure 3:**
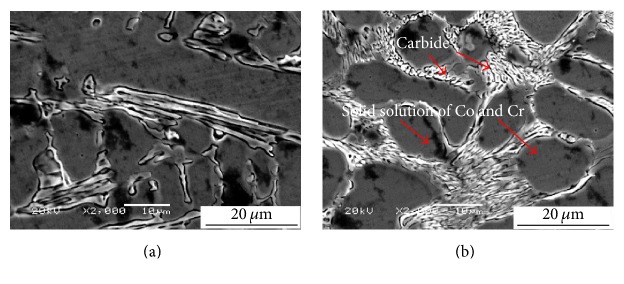
SEM images of the hardfacing layer around the surface of the coating.

The cellular–dendritic carbides are surrounded by the solid solution of Co and Cr, which solidifies toward the surface, as shown in Figure 3. The microstructure of the coating is homogeneous. The dendritic carbides can play a key role in increasing the hardness and wear resistance of the coating. These results are similar to those reported by Xu et al. [26].

### 3.3. Tribological Test of Stellite 6 Alloys

**Figure 4:**
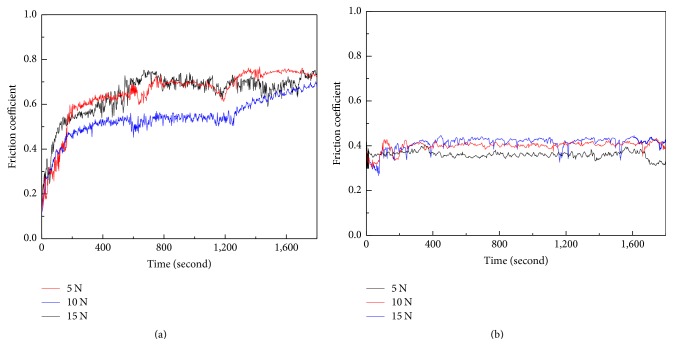
Friction coefficient of Stellite 6 under different loadings (a) at room temperature and (b) at 300°C.

Figure 4 shows the variation of the friction coefficient with sliding time under different loadings. Figures 4(a) and 4(b) indicate that the friction coefficient initially increases rapidly at room temperature and 300°C. With the increase in distance, the wear rate decreases slightly with fluctuation and reaches a stable state [27]. Generally, in the stable state, no noticeable changes in the friction coefficient are observed at low loading.  Figure 4(a) shows that the friction coefficient of Stellite 6 alloys decreases with the increase in loadings. At low loadings, the friction efficient is similar because of the hard phases of Stellite 6 alloy. When the critical value is reached, the friction efficient decreases obviously, as shown in Figure 4(a). The friction coefficient at high temperature increases and reaches the stable state without consuming a considerable amount of time. The values of the friction coefficient at room temperature are higher than those at 300°C because the hardness of the coating decreases and the oxidation of the coating increases [28]. The mechanical properties play a key role in increasing the friction coefficient of the coating because hardness significantly affects the wear performance of Stellite 6 alloy at room and moderate temperatures [29, 30]. At elevated temperature, with the decrease in hardness rate and mechanical properties, the worn surface, with a thin layer and weakly adherent oxide films, is easily destroyed [31, 32]. In such condition, the wear mechanism will be associated with temperature. The wear rate of the coatings can be determined based on the following relation: *K* = *V*/(*F* × *S*), where *F* is the normal load, *S* is the sliding distance, and *V* is the volume of the materials worn out during the test.

**Figure 5:**
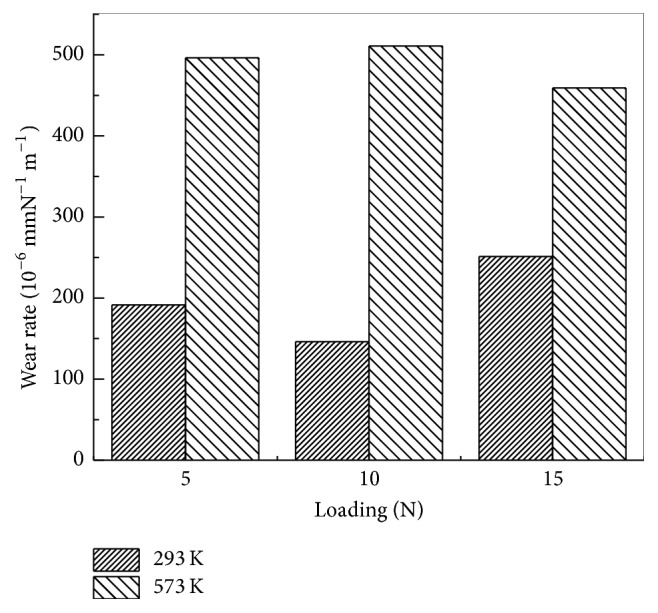
Wear rate of Stellite 6 under different loadings at room temperature and 300°C.

The variations of the wear rate of the coating at room temperature and 300°C under different loadings are shown in Figure 5. With the increase in loadings, the wear rate of Stellite alloy coating increases. The wear rate reaches 250 × 10^−6 ^mm N^−1^·m^−1^ when 15 N is applied to the Stellite 6 alloy coating. However, when applied at 300°C, the wear rate reaches approximately 500 × 10^−6 ^mm·N^−1^·m^−1^ under different loadings. At room temperature, the wear rate is influenced by the loadings. By contrast, at 300°C, the wear rate is relatively stable without any obvious change. These results coincide with those of friction coefficient.

### 3.4. Morphology of Stellite 6 Coating after the Wear Test

**Figure 6:**
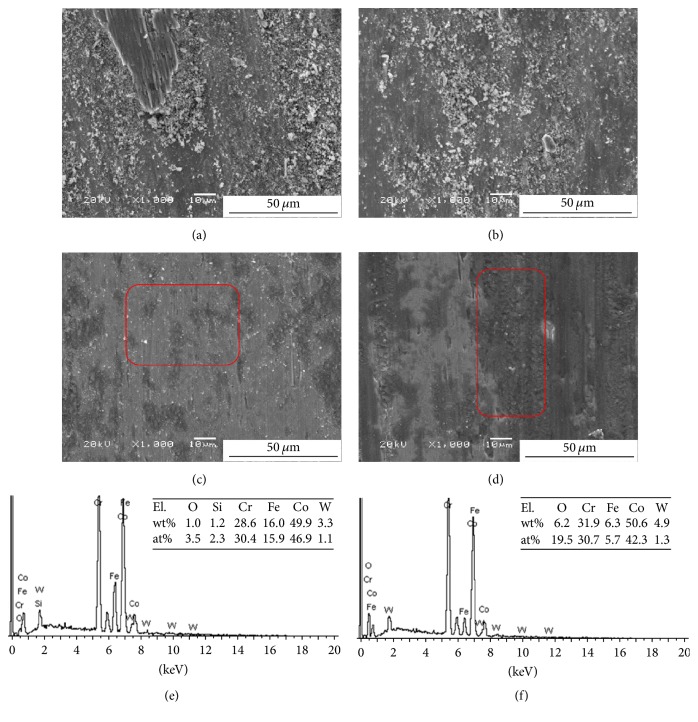
SEM images of Stellite 6 after friction tests at room temperature under (a) 10 N and (b) 15 N applied loadings and at 300°C under (c) 10 N and (d) 15 N applied loadings. (e) EDS analysis of Figure 6(c) and (f) EDS analysis of Figure 6(d).

Figure 6 shows the SEM images of Stellite 6 alloy coating at room temperature and 300°C. Notably, a large number of particles accumulated on the surface of the coating at room temperature compared with the morphology of the coating at 300°C after the tribological test, as shown in Figures 2(a) and 2(b). These particles cannot form a protective layer and cause the increase in the friction coefficient, which accelerates the wear rate. Ploughing scars also appear on the surface of the coating. The friction coefficient of the coating decreases with the increase in the applied loading because of the large number of particles, as shown in Figure 4(a). In this case, abrasive wear is the key mechanism at room temperature.

Figures 6(c) and 6(d) show the smooth morphology of the coating. Wear and plastic deformation during the test are observed from the SEM images of the wear line of the coating. The smooth surface of the wear track is subjected to oxidation at 300°C. The oxide layer can be utilized as a lubricant. Motallebzadeh et al. reported that the wear mechanisms of Stellite 12 alloy are plasticity at 300°C and oxidative wear at 700°C [33]. The friction coefficient at 300°C is lower than that at room temperature, which coincided with the results of wear rate shown in Figure 5. The surfaces exhibit a laminar shape, and no obvious hard precipitates were observed on the surface. In this case, microploughing and plasticity are the key wear mechanisms at 300°C [34, 35]. Figures 6(e) and 6(f) show the energy-dispersive X-ray spectroscopy (EDS) analysis of the worn surface of Stellite 6 alloy at 300°C. The formation of oxides, such as Fe_2_O_3_, CrO, CoO, and Cr_2_O_3_, at elevated temperature is shown in Figures 6(e) and 6(f). The content of the oxidative layer increases significantly with the increase in loadings, which is indicative of more CoO oxidized during the sliding test.

**Figure 7:**
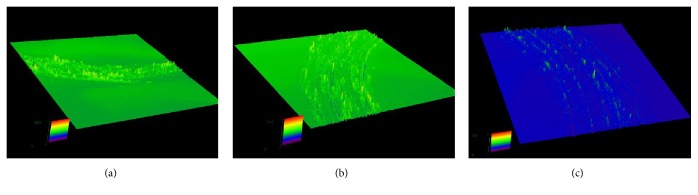
3D morphology of Stellite 6 at high temperature under (a) 5 N, (b) 10 N, and (c) 15 N applied loadings.

Figure 7 shows the 3D morphology of Stellite 6 alloy coating after the tribological test. The worn surface of the coatings shows a large wear track. Many flat planes, which are solid oxide compact layers, are observed along the track. At low loading, many mountain-like embossments are observed on the surface at the applied loading of 15 N. The surface of the coating is coarse after the wear test. The profiles of three specimens under different applied loadings are similar and coincide with the results of wear rate shown in Figure 5.

## 4. Conclusion

The slide wear performances of Stellite 6 alloy coating on Q235 stainless steels at room temperature and 300°C have been compared. The friction coefficient of the coating at room temperature is higher than that at 300°C. With the increase in applied loading, the friction coefficient of Stellite 6 alloy increases at room temperature. However, at 300°C, the friction coefficient of Stellite 6 alloy fluctuates slightly under different applied loadings. The coating after sliding wear at 300°C is subjected to oxidation. The oxide layer can be utilized as a lubricant. The wear rate at 300°C is higher than that at room temperature because of microploughing. Microploughing and plasticity are the key wear mechanisms at 300°C.

## Figures and Tables

**Table 1:** Nominal chemical compositions of Stellite 6 (wt%).

Composition	C	Cr	Si	W	Fe	Mo	Ni	Mn	Co
Mass percentage	1.15	29.00	1.10	4.00	3.00	1.00	3.00	0.50	Bal
